# Female Menstrual Cup Causing Renal Colic, Hydronephrosis, and Ureteral Stricture: A Case Report

**DOI:** 10.5811/cpcem.47383

**Published:** 2025-08-26

**Authors:** Cassidy T Yoshida, Angela Vu, Robert Lam, Sean Donahue

**Affiliations:** * University of Colorado Anschutz Medical Center, School of Medicine, Aurora, Colorado; † Memorial Central Hospital, Department of Emergency Medicine, Colorado Springs, Colorado

**Keywords:** renal colic, menstrual cup, ureteral stricture, hydronephrosis, case report

## Abstract

**Introduction:**

Renal colic is a common reason for patients to present to the emergency department (ED). The most common reasons for this pain are usually renal in origin. Here we present the case of a 45-year-old woman with severe right-sided flank pain and associated hydronephrosis secondary to ureteral obstruction caused by the suction of a menstrual cup.

**Case Report:**

A 45-year-old female presented to the ED with sudden severe right-sided flank pain. The patient endorsed nausea without vomiting, fever, chills, hematuria, or dysuria. She stated that she was currently having her menstrual period. On physical exam, the patient was in distress but had no tenderness with palpation of the flank or abdomen. A computed tomography of the kidneys, ureters, and bladder did not show renal or ureteral stones but demonstrated right-sided hydronephrosis secondary to an anatomical blockage of the ureter, which had been suctioned and involuted into a malpositioned menstrual cup. The patient removed her menstrual cup and had immediate relief of her symptoms. She was observed and remained completely asymptomatic upon reassessment two hours later.

**Conclusion:**

Ureteral obstruction and hydronephrosis is a rare complication of menstrual cup use. As these devices become more common, emergency physicians must be aware of this complication as a cause of severe back pain in menstruating women.

## INTRODUCTION

Renal colic is a common presentation for patients presenting to the emergency department (ED), with approximately 1–2 million cases annually.^1^ The most common cause of this pain is usually renal in origin, with kidney stones and associated pyelonephritis being the main culprits. Additional etiologies for renal colic include perinephric abscesses, mechanical obstruction due to a tumor, or extrinsic blockage of the ureter or bladder. In this case report we present a 45-year-old woman with severe right-sided flank pain and associated hydronephrosis secondary to the suction of a menstrual cup, causing a ureteral obstruction by suctioning her vaginal wall, ureter, and adnexal vasculature into the cup.

## CASE REPORT

A 45-year-old female with no past medical or surgical history presented to the ED with 10/10, sudden-onset, sharp, constant, and non-radiating right-sided flank pain that started three hours prior while she was driving. The patient endorsed nausea without vomiting but denied diarrhea, constipation, fever, chills, hematuria, or dysuria. She had not had pain like this before. The patient stated that she was currently having her menstrual period. She denied any history of kidney stones, trauma, or injury. On physical exam, the patient was in distress due to pain but had no tenderness with palpation of the flank or abdomen.

The differential diagnosis included obstructing kidney stone, infected stone, pyelonephritis, urinary tract infection, and muscle strain. Her laboratory testing showed an unremarkable urinalysis aside from trace blood and a complete blood count with mild leukocytosis showing a white blood cell count of 15 × 10^9^ per liter (L) (reference range: 4.0–11.1 × 10^9^/L). Her basic metabolic panel did not show elevation of creatinine. A computed tomography (CT) of the kidneys, ureters, and bladder did not show renal or ureteral stones but demonstrated right-sided hydronephrosis secondary to an anatomical blockage of the ureter, which had been suctioned and involuted into a malpositioned menstrual cup (Image).

Prior to the CT result, the patient was treated for assumed renal colic due to an obstructing stone. She received intravenous (IV) pain control and IV fluids with minimal relief. After reviewing the CT, we asked her to remove her menstrual cup, which resulted in immediate relief of her symptoms. She was observed and remained completely asymptomatic upon reassessment two hours later.

## DISCUSSION

Renal colic is a common condition that typically presents with severe sudden-onset unilateral flank pain. Initial evaluation of renal colic involves a thorough history, physical exam, and careful consideration of etiology. While nephrolithiasis is a common cause, other etiologies should be considered, particularly in patients with atypical presentations.

*CPC-EM Capsule*

What do we already know about this clinical entity?

*Hydronephrosis causing renal colic is a rare complication of menstrual cup use, with only a few reported cases suggesting this possible but uncommon effect*.

What makes this presentation of disease reportable?

*Although a few cases link menstrual cups to severe renal colic, it is rare and no cases have been reported in any American journals previously*.

What is the major learning point?

*Consider menstrual cups as a rare cause of hydronephrosis in menstruating patients presenting with renal colic, especially when no other cause is found*.

How might this improve emergency medicine practice?

*Raises provider awareness to consider menstrual cup complications in the differential for renal colic in menstruating patients, aiding faster diagnosis and appropriate care*.

In recent years, many new menstrual products such as the menstrual cup or menstrual disc have become a more popular choice for menstruating women given their convenience, cost effectiveness, and decreased ecologic impact.^2^ A study by the Harvard School of Public Health found that 19% of women used menstrual cups.^3^ Menstrual cups are reusable, flexible, bell-shaped, self-retaining, silicone, intravaginal devices that collect menstrual fluid and rely on suction to the vaginal wall to prevent leakage.^4^ Correct placement takes practice while incorrect positioning can have unintended effects such as blood leakage or temporary pelvic discomfort. These symptoms usually resolve with correct placement of the menstrual cup or disc.^5^ Rarely, use of menstrual cups can also cause displacement of an intrauterine device, rashes, or vaginal wounds.^5^,^6^ If pain persists after placement or renal colic ensues, ureteral entrapment may be the cause. Upon literature review, we found that menstrual cup-induced hydronephrosis is a rare complication not previously documented in a journal published in the United States.^5^–^8^

In this case, our patient presented with severe renal colic and associated hydronephrosis secondary to a misplaced menstrual cup. The suction mechanism of menstrual cups, combined with the vaginal wall’s elasticity and the ureters’ proximity, can increase the risk of ureteral obstruction when malpositioned.^7^ This subsequent blockage can cause severe flank pain secondary to unilateral ureteral obstruction and hydronephrosis, which is usually immediately relieved by removal of the menstrual cup.^8^ Given that renal colic is a common reason to visit the ED, menstrual cup entrapment causing hydronephrosis should be considered in menstruating females.

## CONCLUSION

Ureteral obstruction and hydronephrosis is a rare side effect of menstrual cup use. As these devices become more common, emergency physicians must be aware of this complication as a cause of severe back pain in menstruating women.

## Figures and Tables

**Image:**
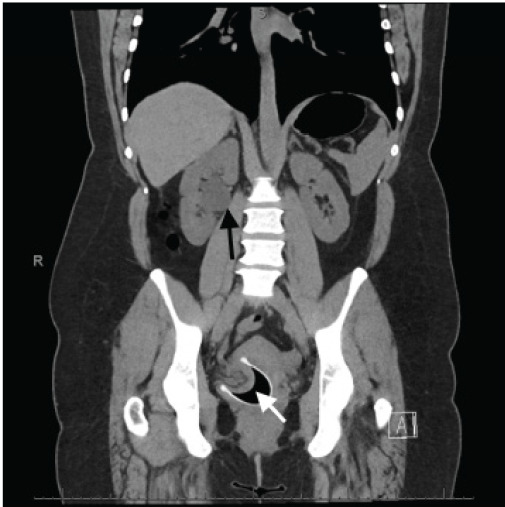
Computed tomography of the kidneys, ureters, and bladder showed mild right hydronephrosis (black arrow) with a transition point at the lower pelvis secondary to a malpositioned menstrual cup with parts of the right vaginal wall, ureter, and adnexal vasculature insinuating into the cup (white arrow).
